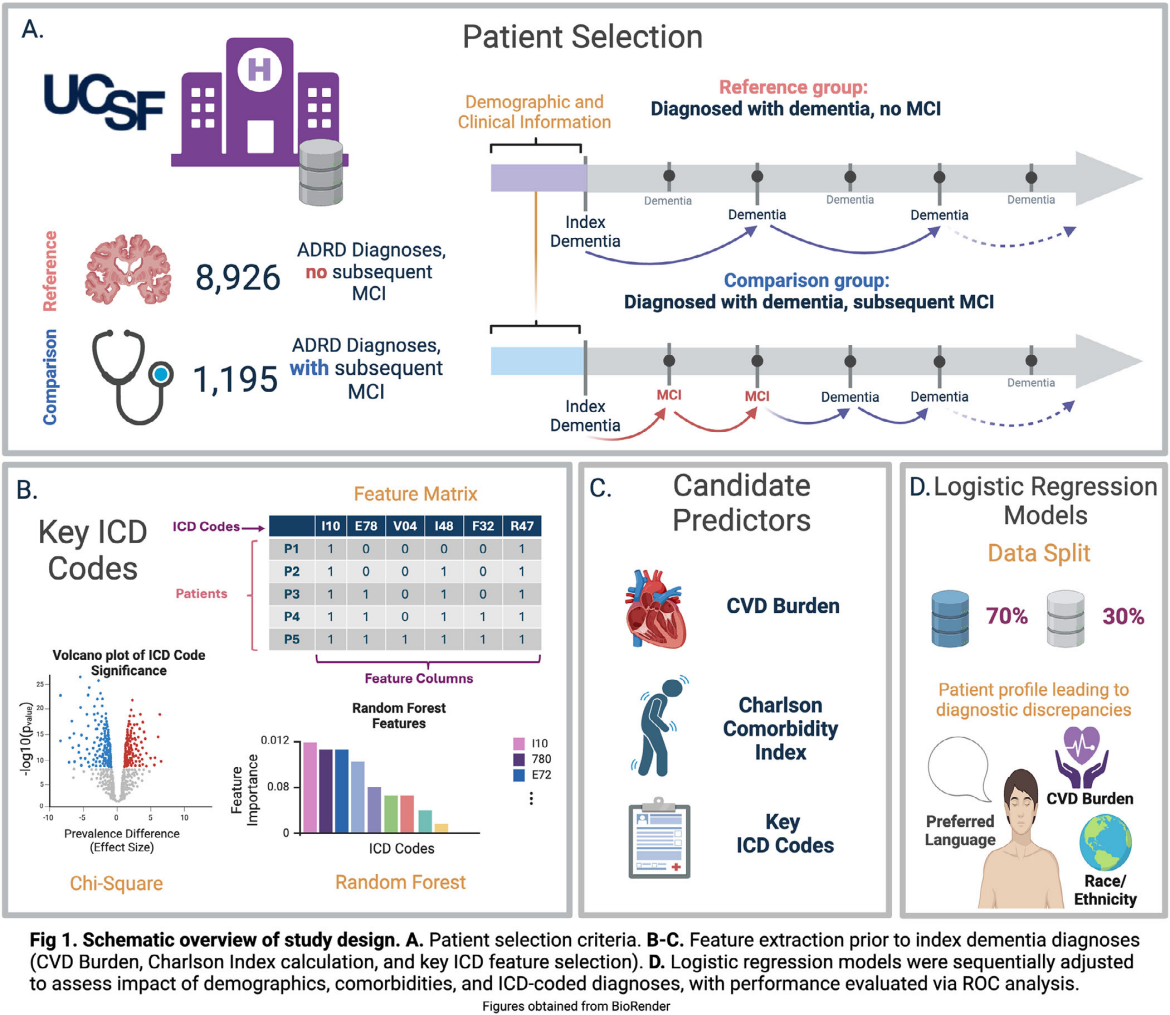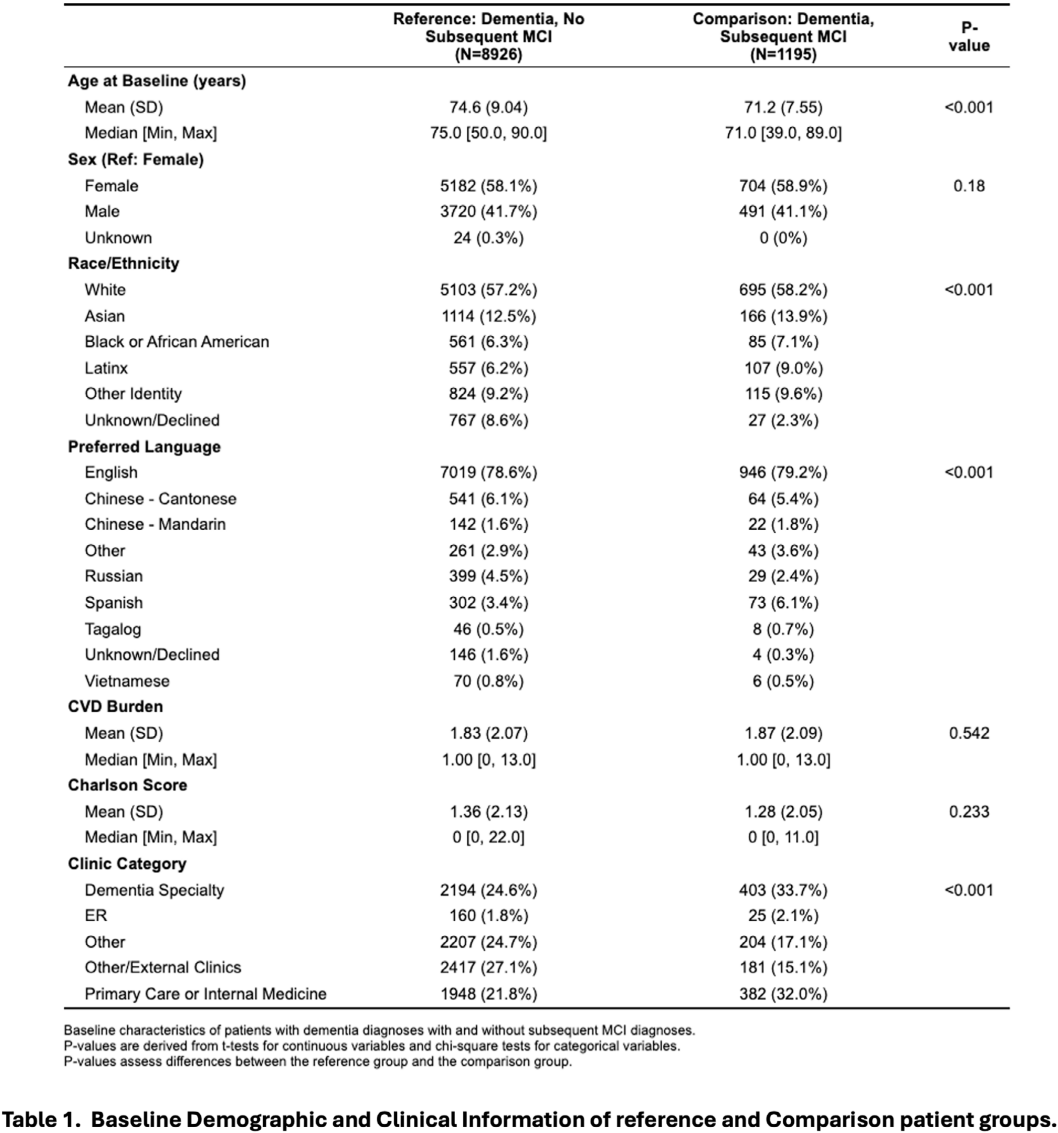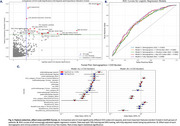# Dementia Prior to MCI? Exploring Frequency and Factors Associated with Diagnostic Discrepancies in a Large EMR System

**DOI:** 10.1002/alz70860_106680

**Published:** 2025-12-23

**Authors:** Silvia Miramontes, Hunter Mills, Boris Oskotsky, Evan Phelps, M. Maria Glymour, Marina Sirota, Elena Tsoy

**Affiliations:** ^1^ Bakar Computational Health Sciences Institute, University of California, San Francisco, San Francisco, CA, USA; ^2^ University of California, San Francisco, San Francisco, CA, USA; ^3^ Boston University School of Public Health, Boston, MA, USA; ^4^ Memory and Aging Center, University of California San Francisco, San Francisco, CA, USA; ^5^ Global Brain Health Institute, University of California, San Francisco, San Francisco, CA, USA

## Abstract

**Background:**

Mild cognitive impairment (MCI) is an early stage of cognitive decline, typically preceding dementia. However, the temporal order of these diagnostic categories in real‐world clinical practice may be reversed, having significant clinical implications for patient care and family burden. This study investigates demographic and clinical predictors of these diagnostic discrepancies within a large metropolitan healthcare system.

**Method:**

We categorized patients aged 50+ who received a dementia diagnosis at UCSF Health Center (*N* = 10,121, Table 1) into two groups: the reference group (diagnosed with incident dementia and no subsequent MCI diagnosis, *N* = 8,926) and the diagnostic discrepancy group (diagnosed with incident dementia and subsequent MCI, *N* = 1,195). Logistic regression models estimated associations of demographic (sex, age, race/ethnicity, preferred language), clinical (CVD Burden, Charlson Comorbidity Index, and key ICD codes identified via chi‐square and random forest models), and care setting (clinic specialty) factors with diagnostic discrepancies between cohorts (Figure 1).

**Result:**

Patients were more likely to experience diagnostic discrepancies if they were younger (OR=1.04 per year of age, 95%CI: 1.03‐1.05), were Spanish‐speaking (OR=1.75, 95%CI: 1.37–2.24), had higher CVD burden (OR = 1.07, 95% CI: 1.04–1.10), or were initially diagnosed in primary care/internal medicine settings (OR = 1.28, 95% CI: 1.09–1.49). ICD codes linked to higher odds of experiencing diagnostic discrepancies included dizziness (OR = 1.35, 95% CI: 1.10–1.65), gait abnormalities (OR = 1.32, 95% CI: 1.08–1.62), and general symptoms (e.g., fever) (OR = 1.68, 95% CI: 1.42–1.99; Figure 2). AUC‐ROC from fully adjusted models was 0.698.

**Conclusion:**

This study identified demographic, clinical, and care setting variables associated with diagnostic discrepancies in a large patient population at a metropolitan health center. Our results emphasize the importance of continued efforts to understand and address inequities in diagnostic pathways, which are especially relevant in the current era of disease‐modifying treatments targeting early disease processes. We will discuss the implications of these findings for clinical care and the development of targeted interventions for improved and equitable diagnostic workflows.